# Beta-Adrenergic Modulation of Tremor and Corticomuscular Coherence in Humans

**DOI:** 10.1371/journal.pone.0049088

**Published:** 2012-11-20

**Authors:** Mark R. Baker, Stuart N. Baker

**Affiliations:** Institute of Neuroscience, Newcastle University, Framlington Place, Newcastle upon Tyne, United Kingdom; University College London, United Kingdom

## Abstract

Coherence between the bioelectric activity of sensorimotor cortex and contralateral muscles can be observed around 20 Hz. By contrast, physiological tremor has a dominant frequency around 10 Hz. Although tremor has multiple sources, it is partly central in origin, reflecting a component of motoneuron discharge at this frequency. The motoneuron response to ∼20 Hz descending input could be altered by non-linear interactions with ∼10 Hz motoneuron firing. We investigated this further in eight healthy human subjects by testing the effects of the beta-adrenergic agents propranolol (non-selective β-antagonist) and salbutamol (β_2_-agonist), which are known to alter the size of physiological tremor. Corticomuscular coherence was assessed during an auxotonic precision grip task; tremor was quantified using accelerometry during index finger extension. Experiments with propranolol used a double-blind, placebo-controlled crossover design. A single oral dose of propranolol (40 mg) significantly increased beta band (15.3–32.2 Hz) corticomuscular coherence compared with placebo, but reduced tremor in the 6.2–11.9 Hz range. Salbutamol (2.5 mg) was administered by inhalation. Whilst salbutamol significantly increased tremor amplitude as expected, it did not change corticomuscular coherence. The opposite direction of the effects of propranolol on corticomuscular coherence and tremor, and the fact that salbutamol enhances tremor but does not affect coherence, implies that the magnitude of corticomuscular coherence is little influenced by non-linear interactions with 10 Hz oscillations in motoneurons or the periphery. Instead, we suggest that propranolol and salbutamol may affect both tremor and corticomuscular coherence partly via a central site of action.

## Introduction

Many studies have investigated oscillatory coupling between the sensorimotor cortex and the periphery using coherence analysis. During steady contractions, cortical recordings (electroencephalogram, EEG, or magnetoencephalography, MEG, in humans, local field potentials in animals) show coherence with contralateral rectified electromyogram (EMG) at frequencies of 15–30 Hz [1]–[6]. Both efferent and afferent (feedback) pathways contribute to this oscillatory coupling [7]–[11].

In the periphery, oscillations are greatest at ∼10 Hz. Physiological tremor has a strong component around this frequency, probably as a result of multiple interacting factors. These include mechanical resonance at skeletal articulations [12], the stretch reflex loop [13], motor unit recruitment [14] and motor unit synchronisation [15]. A component of ∼10 Hz physiological tremor is centrally generated [15]–[17]. Although both cortical and muscle recordings show oscillations around 10 Hz and 20 Hz, significant corticomuscular coherence is usually only seen for the higher frequency band, despite ∼10 Hz oscillations being effectively carried from the motor cortex down the corticospinal tract [18]. We have recently provided evidence that neural circuits in the spinal cord phase invert signals around 10 Hz [19], [20]. Convergence of in-phase and out-of-phase signals on motoneurons results in phase cancellation, reducing the amplitude of signals at frequencies related to physiological tremor. This may have functional consequences in improving movement precision.

The non-linear nature of spinal circuits means that interactions can occur across different frequency bands. For example, our previous computational modelling suggested that recurrent inhibition by Renshaw cells will boost signals around 30 Hz, at the same time as reducing those around 10 Hz, leading to a reciprocal modulation of power at these frequencies [19]. Conversely, the highly periodic nature of motoneuron firing at low forces can produce oscillations in motor output, even when the synaptic input to motoneurons is not oscillatory [19]. As the firing of the motoneuron pool becomes more or less periodic, this effect will mean that the amplitude of oscillations at 10 Hz and 20 Hz tends to co-modulate. We were interested to examine experimentally how motor systems oscillations at different frequencies might interact, since this could tease apart the different factors influencing oscillatory amplitude and coupling.

One approach to this issue is to manipulate the system pharmacologically. We have previously shown that the benzodiazepine diazepam modulates cortical oscillations in the beta band, without altering coupling to the periphery [21]. By contrast, the anti-epileptic drug carbamazepine increases beta band corticomuscular coherence, without affecting the amplitude of cortical oscillations [22]. However, neither of these agents affected oscillations around 10 Hz. Clinically, the most powerful effects on physiological tremor are produced by beta-adrenergic agents. The beta-adrenergic agonists enhance tremor (adrenaline [23]; isoprenaline [24]; salbutamol [25]), whereas the antagonist propranolol reduces it [24]. These drugs are generally believed to act by changing the gain of muscle receptors and spinal reflex loops, mediated via peripherally located β_2_-adrenergic receptors [23], [24], [26]–[28]. If the effects of beta-adrenergic agents are solely peripheral, they will change the gain of peripheral feedback, which would inject oscillations into motoneuron firing. This might co-modulate oscillations at 10 Hz and 20 Hz [19].

In this paper, we test this idea directly. Surprisingly, we show that propranolol increases ∼20 Hz corticomuscular coherence but reduces tremor at ∼10 Hz, and that salbutamol has no significant effect on ∼20 Hz corticomuscular coherence, but markedly increases ∼10 Hz tremor. These opposite effects on oscillations at 10 Hz and higher frequencies are similar to those seen in a computational model of recurrent feedback by Renshaw cells [29], and are not expected if these agents only act peripherally. We speculate that beta-adrenergic agents also have a central action, allowing opposite effects on physiological tremor and beta-band corticomuscular coherence.

## Methods

Experiments were performed on 8 young healthy right-handed volunteer subjects (3 female; age range 21–32 years) without a family history of essential tremor. None of the subjects were taking prescription medication either regularly or as required. Subjects were not pre-selected based on their corticomuscular coherence. None of the subjects had received a prior diagnosis of enhanced physiological tremor or had evidence of enhanced physiological tremor when assessed by a clinical neurologist (MRB) at the time of experiments.

### Ethics statement

Informed written consent was obtained in accordance with the Declaration of Helsinki and all procedures were approved by the Cambridge Local Research Ethics Committee.

### Electrophysiological Recordings

Surface EMGs from the right upper limb were recorded with adhesive electrodes (Biotrace 0713C, MSB, Marlborough, UK). Recordings were made from first dorsal interosseous (1DI), abductor pollicis brevis (AbPB), abductor digiti minimi (AbDM), flexor digitorum superficialis (FDS), and extensor digitorum communis (EDC), with an inter-electrode distance of 1.5–2 cm. Differential EEG was recorded from the left sensorimotor cortex using two adhesive scalp electrodes (Neuroline 720 00-S, Medicotest, St Ives, UK) placed 30 mm lateral to the vertex and 20 mm anterior and posterior to the interaural line. The anterior EEG electrode was connected to the non-inverting input of the amplifier; this is the same montage as used in our previous work [7], [11], [21], [22], [30]. Signals were amplified (EMG gain 500–5000; EEG gain 50 k) and bandpass filtered (EMG 30 Hz-2 kHz; EEG 3 Hz-2 kHz) before being digitised at 4273.5 Hz by a Power1401 interface (Cambridge Electronic Design Ltd, Cambridge, UK) connected to a computer running Spike2 software (Cambridge Electronic Design Ltd).

### Measurement of Tremor

Physiological tremor was quantified using accelerometry. A low mass splint was taped to the subject's right index finger, and restricted movements to the metacarpophalangeal joint. Subjects gripped a fixed vertical pole with the thumb and digits 3–5, and extended the index finger. A miniature accelerometer (Isotron 25B, Endevco, San Juan Capistrano, CA, USA) was fixed to the end of the splint, with its sensitive axis aligned to detect finger flexion/extension movements. Recordings were made for 90 s.

### Precision Grip Task

Measurement of corticomuscular coherence was carried out using an auxotonic precision grip task. Subjects held the two levers of a purpose-built manipulandum between thumb and index finger. The aluminium levers (20×80×1.5 mm) were attached to the shafts of two computer-controlled torque motors, which incorporated optical encoders for position measurement. At rest the levers were separated by 70 mm; a 1 N force was required to move the levers off their end-stops.

**Figure 1:**
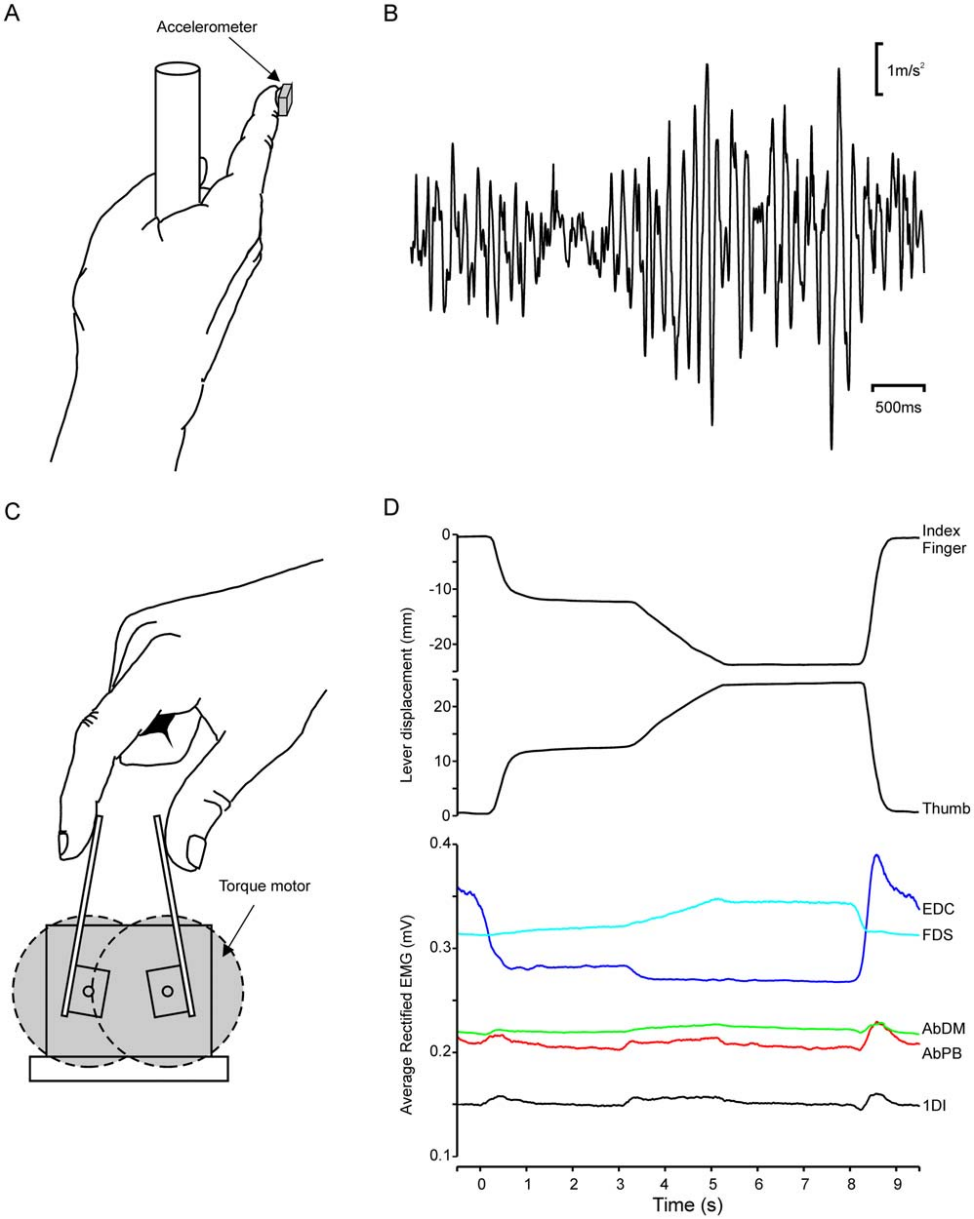
Experimental tasks. A. Postural tremor task. B. Example of postural physiological tremor in a single subject, recorded via an accelerometer. C. Auxotonic precision grip task. D. Lever displacement and average rectified EMG recorded from *extensor digitorum communis* (EDC), *flexor digitorum supeficialis* (FDS), *abductor digiti minimi* (AbDM), *abductor pollicis brevis* (AbPB) and *first dorsal interosseous* (1DI) muscles in a single subject during a single trial of the auxotonic task.

The task (see Figure 1) required subjects to maintain cursors representing each lever position within two moving target boxes on a computer screen. Movement of the levers was resisted by the torque motors, which simulated an auxotonic (spring-like) load. The target boxes produced a hold-ramp-hold pattern, with the first hold requiring a rapid lever displacement of 12 mm from rest followed by a hold period of 3 s at a force level of 1.3 N. The targets then produced a 2 s ramp movement to reach the second hold, with a displacement of 24 mm, 1.6 N force and duration of 3 s, before subjects released the levers. This is the COMP1 task of Kilner et al. [6], and has been used in several of our previous publications [7], [11], [21], [22], [30].

### Experimental Protocol and Drug Administration

Drugs were prescribed and administered by one of the authors (MRB), who is a clinical neurologist. Potential adverse reactions were minimised by using the lowest therapeutic dose recommended by the Joint Formulary Committee [31].

Propranolol experiments were conducted as a double blind randomised placebo-controlled trial. For each subject, placebo and propranolol (40 mg of active compound mixed with vehicle compound), which were indistinguishable in appearance, were randomly assigned a label A or B by an independent randomizer. Each subject participated in experiments on two days, separated by a 2–3 week washout period. At the start of the morning session of each day, the subject was prepared for recording. A control experiment was then carried out, which consisted of 80 trials of the precision grip task and a measurement of tremor. After this, the electrode locations were marked on the skin with ink and the subject ingested experimental compound A (first day) or B (second day). Four hours later, when peak serum concentrations of the active agent should have been achieved [32], the subject returned to the laboratory. Fresh adhesive electrodes were applied at the marked muscle and scalp locations, and further recordings were made. The identities of compound A and B for each subject were only revealed by the randomizer once all experiments were complete and data were ready for analysis. By using a placebo, we were able to control for effects caused by the different time of day of the experimental and control recording sessions, which we have previously shown to be important [22].

The subjects who had participated in the propranolol experiments were also used in the salbutamol experiments, following a washout period of at least one week. After the control recordings, 2.5 mg of salbutamol was inhaled by the subject as an aerosolised solution produced by a nebuliser. Inhaled aerosolised salbutamol is absorbed rapidly via the lungs into the bloodstream. Maximum serum concentrations are achieved ∼10 minutes after inhalation [33]. Nebulised salbutamol, at a dose of 40 µg/kg, results in an average serum concentration of 2.5 ng/ml after inhalation [34]. The rapid absorption meant that control and test experiments were separated by only a short period, and we accordingly considered it unnecessary to carry out placebo experiments for salbutamol (see paragraph 12 of the Results section for further justification).

Electrocardiogram (ECG) was monitored throughout the salbutamol experiments. Salbutamol at an average plasma concentration of 2.5 ng/ml produces a 10% increase in heart rate above baseline [34]. The second recording session (after salbutamol) was therefore only commenced after the heart rate had increased by 10% relative to baseline.

### Analysis

All data analysis was performed using custom written MATLAB (Mathworks Inc.) routines, and followed the procedures used in our prior publications [7], [22]. Prior to analysis EMG recordings were full wave rectified.

Analysis of tremor power used 0.88 s long (4096 points for a sampling rate of 4630 Hz) sequential non-overlapping data sections, taken from the entire available duration of the accelerometer recording. These were processed with a Fast Fourier Transform, yielding a tremor power spectrum with frequency resolution of 1.13 Hz.

Analysis for the precision grip task focused upon the second hold phase, when ∼20 Hz corticomuscular coherence is greatest [6]. EEG and EMG power spectra, and coherence between EEG and EMG, were computed using three contiguous 0.88 s-long sections of data taken from this task phase of each trial, and a 4096-point-long Fast Fourier Transform [3].

Accelerometer, EEG and EMG power spectra were first normalised to the total power in that signal across all frequencies. For acceleration, the normalisation used the total power in the first recording of that day, allowing overall changes in tremor power to be measured. For EEG and EMG, each spectrum was normalised to its own total power, revealing the magnitude of spectral peaks relative to the total. The spectra were then averaged across eight subjects for EEG or acceleration, and across eight subjects and five muscles for EMG. Coherence spectra were similarly averaged across subjects and muscles; significance limits for the averaged coherence were calculated according to the method described in Evans and Baker [35] and Baker et al [8].

Power was summed across a frequency band of interest (6.2–11.9 Hz for tremor; 15.3–32.2 Hz for beta-band oscillations) within a single subject. Changes of power before and after substance administration in these frequency bands were then assessed using paired t-tests (P<0.05). For propranolol, we needed to test not only whether propranolol produced a change, but whether this change was significantly different from that produced by placebo. Accordingly, the change in power in the relevant band was found for each subject; these changes were compared between placebo and propranolol (paired t-test, P<0.05).

For corticomuscular coherence, coherence was averaged across muscles and across bins within the 15.3–32.2 Hz band for each subject. The significance of the changes in coherence before and after substance administration was determined by finding
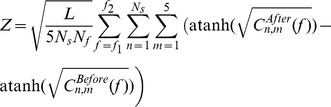
(1)Where L is the number of disjoint sections used to calculate the coherence, and *C^After^_n,m_(f)* and *C^Before^_n,m_(f)* are the coherence calculated at frequency bin *f* for subject *n*, muscle *m* before and after substance administration. *Z* is computed from a sum over all available *N_s_* subjects, *N_f_* frequencies (*N_f_ = f_2_−f_1_+1*) and 5 muscles. On the null hypothesis that coherence is the same before and after substance administration, Z will be normally distributed with zero mean and unit variance [21], [30]. The significance of changes in coherence was thus determined with reference to values of the standard normal probability distribution.

In experiments investigating the effects of propranolol, it was necessary to compare the changes caused by propranolol with those seen following placebo. To achieve this, we therefore calculated:
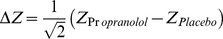
(2)The normalisation by 1/√2 once again ensured that, on the null hypothesis that the substances cause equal changes in coherence, ΔZ will be normally distributed with zero mean and unit variance.

We determined the effect of a drug or placebo on the coherence phase as follows. Firstly, frequency bins in the beta-band region (15.3–32.2 Hz) for each muscle were identified which had significant coherence both before and after substance administration. The coherence phase was measured for those bins, and the difference in phase determined between the ‘before’ and ‘after’ conditions. The circular average of this difference was computed [36]. We then randomly shuffled the individual paired phase measurements, reallocating each member of a pair to the ‘before’ or ‘after’ categories; the circular average of the phase differences was recalculated. The shuffling procedure was repeated 10^5^ times, allowing estimation of the distribution of the circular mean phase difference, on the null hypothesis that there was no change before and after substance administration. If the absolute value of the circular average phase difference from the actual experimental data was larger than *n* of the absolute values of the circular average phase differences determined from the shuffled data, this yielded an approximate Monte Carlo significance level of *P*<*n*/10^5^.

## Results

**Figure 2:**
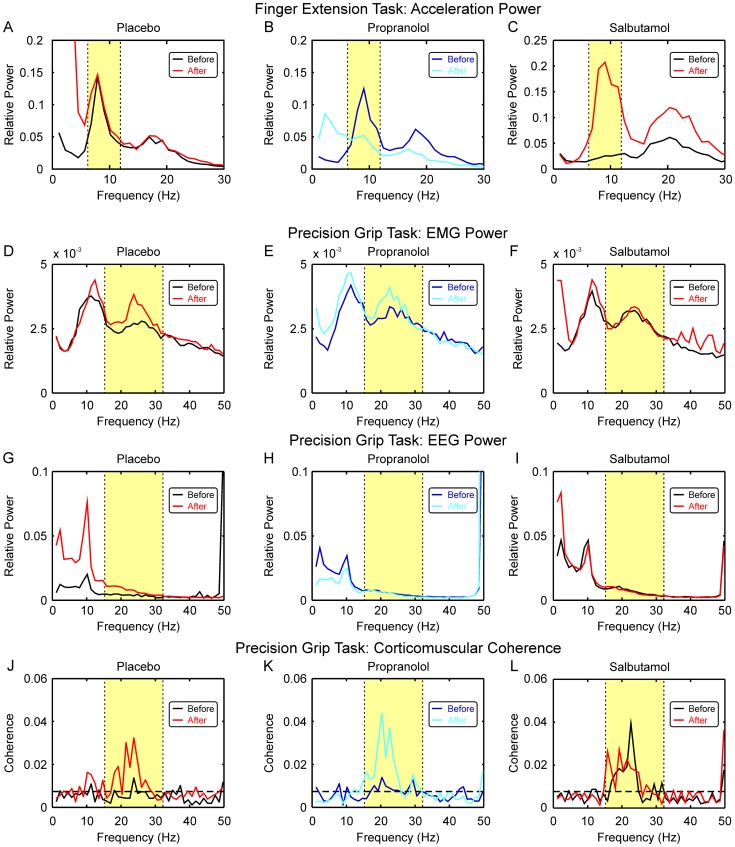
Single Subject Data. Each column of this figure refers to the effects of a different substance: placebo (A,D,G,J), propranolol (B,E,H,K) and salbutamol (C,F,I,L). A–C, effect on postural tremor during an index finger extension task. Traces show the power spectrum of the finger acceleration, before and after substance ingestion. Yellow shaded region corresponds to the band of physiological tremor (6.2–11.9 Hz). D–F, effect on EMG power. G–I, effect on EEG power. J–L, effect on corticomuscular coherence. In (D–L), all measurements were made during the second hold phase of a hold-ramp-hold auxotonic precision grip task (see Methods). Yellow shaded region marks the 15.3–32.2 Hz band of beta oscillations. EMG power spectra (D–F) and corticomuscular coherence (J–L) have been averaged over all five muscles recorded. In (J–L), horizontal dashed lines indicate the significance level for the averaged coherence.

Figure 2 illustrates the results obtained from a single subject in each experimental protocol tested. Figures 2A–C show the effect of the different substances administered on physiological tremor, measured from the acceleration power spectrum during finger extension. In this subject, two peaks were visible in the tremor spectra, around 8 Hz and around 20 Hz; the ∼10 Hz region usually associated with physiological tremor is marked with yellow shading. Each panel of Fig. 2A–C relates to measurements made on a different day; it is notable that there was high day-to-day variability in the baseline tremor levels (compare Fig. 2C with 2A&B). There was little change in tremor following the placebo (Fig. 2A), but a substantial reduction after propranolol (Fig. 2B) in this subject. By contrast, salbutamol dramatically increased tremor (Fig. 2C).

The remaining panels of Fig. 2 show results from the auxotonic precision grip task, which was used to examine beta-band oscillations in the motor system. Accordingly, the yellow shading in these plots highlights the 15.3–32.2 Hz band, which encompassed the beta peaks. In this subject, there was a small increase in the size of the power spectral peak at these frequencies in the EMG following both placebo and propranolol (Fig. 2DE); this is probably attributable to the time of day of the recordings, as the two datasets were gathered four hours apart to allow time for absorption of the orally administered substance. By contrast, salbutamol appeared to have little effect on the beta band peak in the EMG (Fig. 2F). The placebo recordings in this subject showed a non-specific rise in EEG power across many frequencies (Fig. 2G), but neither propranolol nor salbutamol seemed to affect the beta-band EEG peak (Fig. 2HI).

In the initial recordings for placebo and propranolol administration (Fig. 2JK), this subject showed weak beta-band corticomuscular coherence which barely rose above the significance level (dashed lines). In both cases, the coherence peak was enhanced in the second recording of the day. The control recording for salbutamol showed clearer coherence (Fig. 2L), but this did not change consistently following salbutamol inhalation. The high day-to-day variability in baseline measures is again of note; we have previously reported fluctuations in corticomuscular coherence from single subjects both diurnally [22] and over longer timescales [11]. Diurnal changes in coherence probably explain the apparent effect of placebo in Fig. 2J: we have previously shown that coherence increases in measurements made later in the day [22].

**Figure 3:**
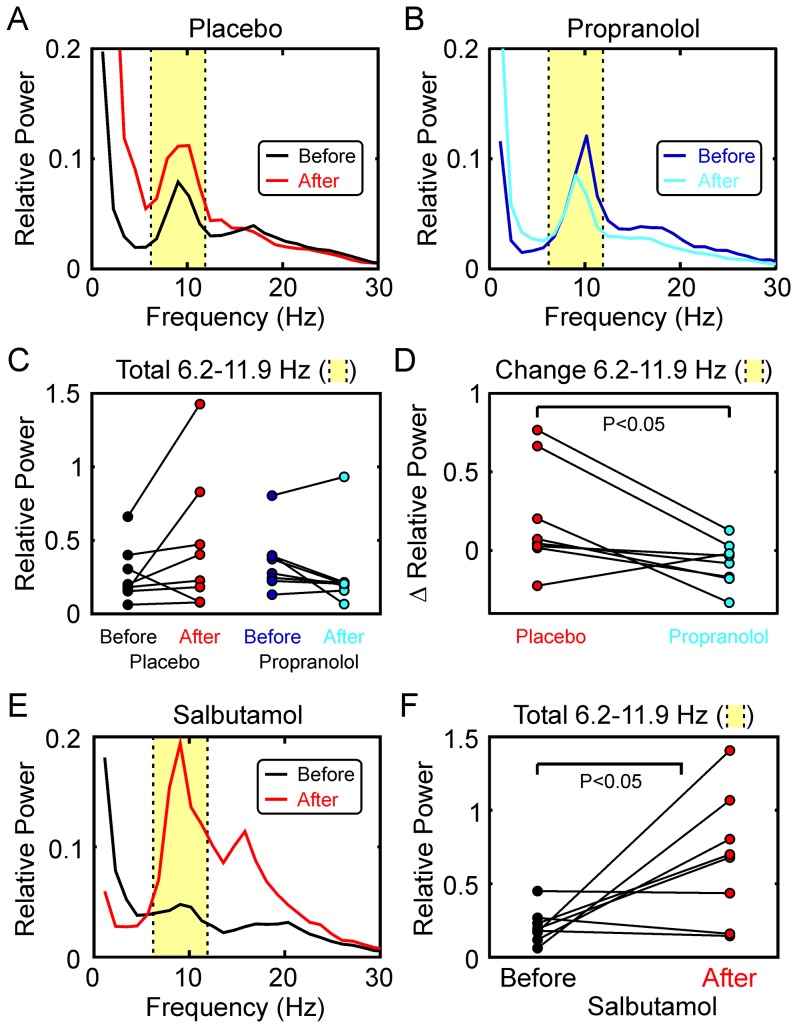
Effects on Resting Tremor. All measurements in this figure were taken during index finger extension. A, B, changes in finger acceleration power spectra, averaged across all eight subjects, before and after administration of placebo (A) and propranolol (B). C, total acceleration power over the 6.2–11.9 Hz range (yellow shading in A,B) before and after substance administration. Each point shows data from one subject; lines link data from the same subject. Data points are colour coded to match the colours used in (A,B). There was no significant change in tremor power after either placebo or propranolol. D, changes in tremor power after administration of placebo or propranolol. Each point shows the difference between the ‘after’ and ‘before’ points from the corresponding subject shown in (C). Propranolol reduced tremor power significantly compared to placebo (P<0.05). E, changes in finger acceleration power spectra, averaged across all eight subjects, before and after administration of salbutamol. F, total acceleration power over the 6.2–11.9 Hz range (yellow shading in E) before and after salbutamol administration. Tremor power was significantly increased after salbutamol (P<0.05).

Figure 3 shows population data on the effects of the two drugs tested on physiological tremor. Figure 3AB plots the tremor power spectra, averaged across all eight subjects. There appear to be some changes in the peak around 10 Hz, with an increase in the tremor following placebo, but a reduction following propranolol. Figure 3C shows how the average power over the tremor band (6.2–11.9 Hz) changed in individual subjects. Although there was a trend for increased tremor with placebo, and decreased tremor with propranolol, these changes were not significant. Figure 3D presents individual data on the change in tremor power following each substance. The difference in tremor was smaller for propranolol than for placebo in 7/8 subjects. This change was significantly different: propranolol reduced tremor compared with placebo (P<0.05, paired t-test and also binomial test).

Figure 3E shows averaged power spectra of finger acceleration before and after the inhalation of salbutamol. As with the single subject illustrated in Fig. 2C, salbutamol markedly increased the average tremor peak. The changes seen in individual subjects are presented in Fig. 3F; salbutamol significantly increased tremor power across the subject population.

These results confirm that – at the doses used here – propranolol reduces, but salbutamol increases, physiological tremor, which is in accord with previous work [24] and also common clinical experience.

**Figure 4:**
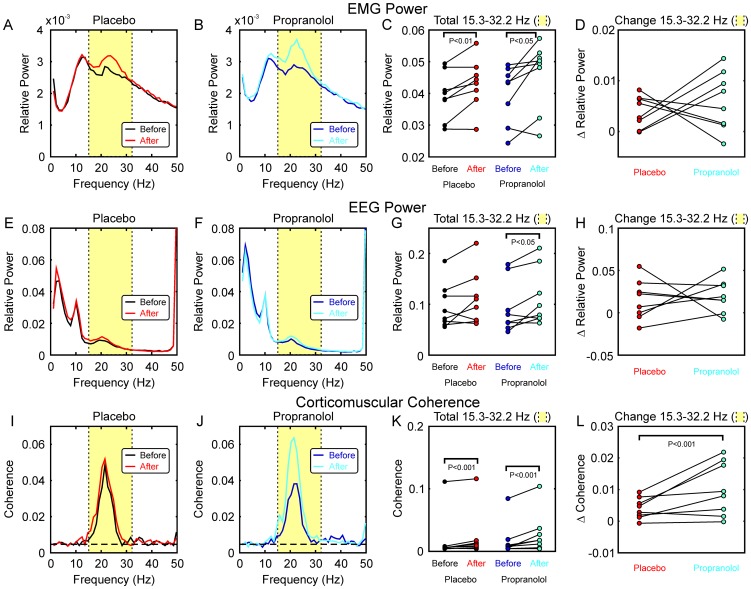
Effects of Propranolol on Beta-band Oscillations during the Auxotonic Precision Grip Task. A,B, EMG power spectra, averaged across all eight subjects and five muscles, before and after administration of placebo (A) and propranolol (B). C, total EMG power in the 15.3–32.2 Hz beta band (shaded yellow in A,B), plotted for single subjects before and after placebo and propranolol. Both substances significantly increased EMG beta power. D, changes in EMG power in the 15.3–32.2 Hz band produced by placebo and propranolol. Each point is the difference of the ‘after’ and ‘before’ points from the corresponding subject in (C). There was no significant difference in the change in beta-band power between placebo and propranolol. E–H, similar display as (A–D), but for EEG power. Propranolol significantly increased EEG power in the beta band, but there was no significant difference between the changes seen after placebo and propranolol. I–L, similar display as (A–D), but for corticomuscular coherence (averaged over all five available muscles). Dashed horizontal lines in (I,J) mark the significance level for the averaged coherence (P<0.05). Coherence was significantly increased after both placebo and propranolol (K), but the change in coherence was significantly greater for propranolol than for placebo (L).

Figure 4 presents the effects of propranolol on oscillations during the second hold phase of the precision grip task. Figure 4AB shows the EMG power spectra, averaged over all eight subjects and five muscles. In both cases, the recordings made later in the day showed a more pronounced beta-band spectral peak. Figure 4C shows individual measures of beta-band power; there were significant increases following both propranolol and placebo administration. However, a comparison of the changes in power (Fig. 4D) revealed no significant difference between the effects of propranolol and placebo.

Using the same format, Fig. 4E–H presents the changes in EEG power following placebo and propranolol administration. In both cases there was a slight increase in the beta-band power peak, which became significant for propranolol (Fig. 4G). However, as for the EMG power, there were no significant differences between the effects of propranolol and placebo.

For both EEG and EMG power we normalised values in a given frequency bin relative to the total power, summed across all frequencies. We also verified that there were no significant changes in the total power, for any of the comparisons described by Fig. 4CDGH.

Finally, Fig. 4I–L shows how corticomuscular coherence altered following ingestion of these substances. There was a very small increase in coherence following placebo, but a more pronounced increase following propranolol (Fig. 4IJ); both of these effects reached significance (Fig. 4K). Propranolol increased beta-band corticomuscular coherence more than placebo (Fig. 4L) in 7/8 subjects, significantly so (using an individual difference of coherence test) in 3 subjects. At a population level, the increase following propranolol was significantly greater than after placebo using the difference of coherence test described in Methods (P<0.001), and also on a binomial test (P<0.05).

**Figure 5:**
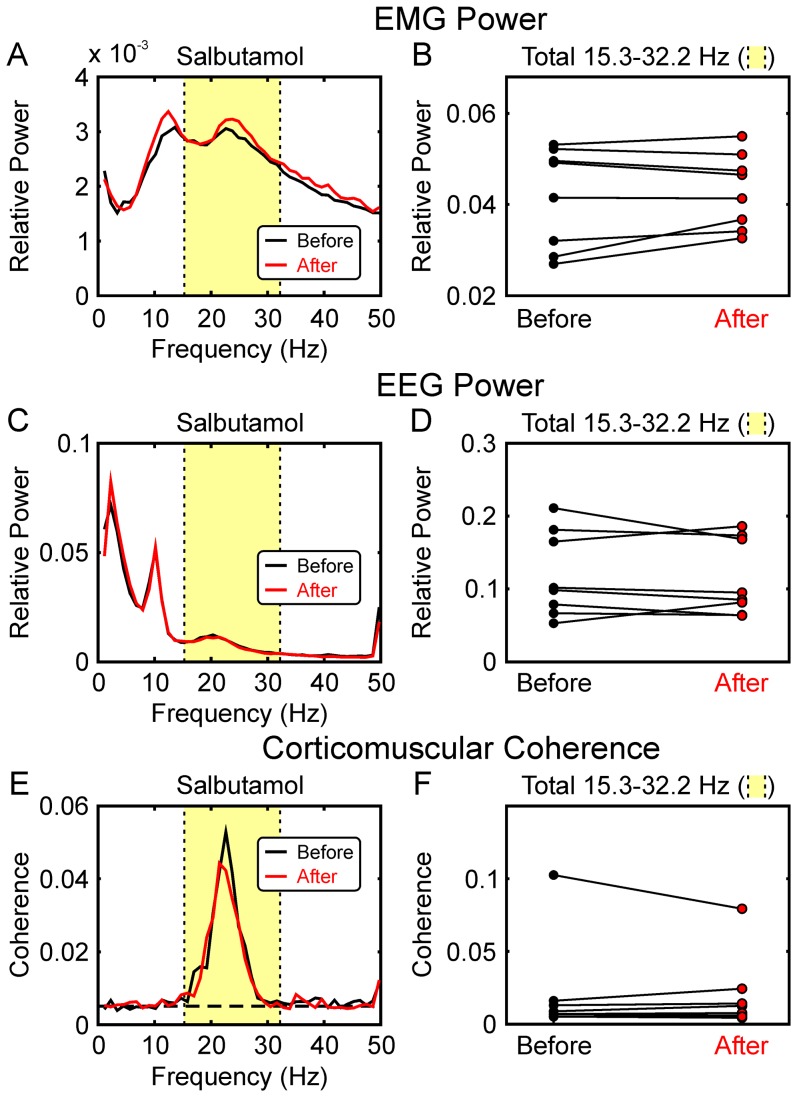
Effects of Salbutamol on Beta-band Oscillations during the Auxotonic Precision Grip Task. A, EMG power spectrum, averaged over all five muscles and eight subjects, before and after administration of salbutamol. B, total power in the 15.3–32.2 Hz band (shaded yellow in A) before and after salbutamol inhalation. Each point gives data from a single subject. C,D, as (A,B), but for EEG power. E,F, as (A,B), but for corticomuscular coherence. Dashed horizontal line in (E) marks the significance limit for the coherence (P<0.05). None of the measures displayed in (B,D,F) significantly changed after salbutamol administration (P>0.05).

The results from the precision grip task following salbutamol inhalation are shown in Fig. 5. There were small or negligible changes in the averaged beta band power spectral peaks in both EMG and EEG signals (Fig. 5AC); these were not consistent across subjects (Fig. 5BD) and not significant. Similarly, the averaged corticomuscular coherence decreased slightly (Fig. 5E), but this was not significant (Fig. 5F).

**Figure 6:**
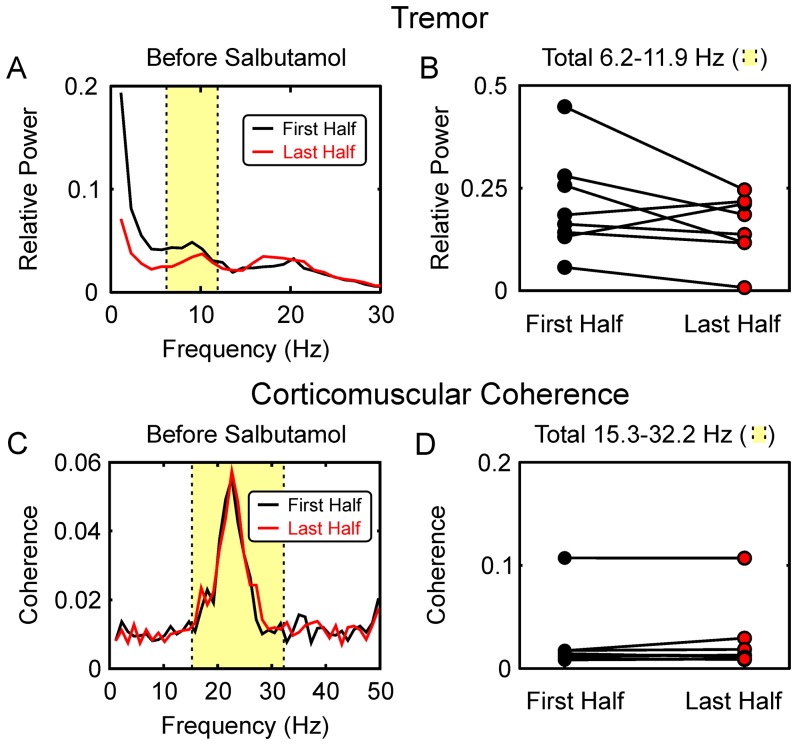
Reproducibility of Tremor and Corticomuscular Coherence. A, finger acceleration power spectra, measured prior to salbutamol administration, averaged across all eight subjects. The available recording from each subject was divided in half, and each half analysed separately. B, total acceleration power over the 6.2–11.9 Hz range (yellow shading in A) in each half of the recording. Each point shows data from one subject; lines link data from the same subject. Data points are colour coded to match the colours used in (A). C, corticomuscular coherence, averaged over all five muscles and eight subjects, measured from the first and last half of the available recording in each subject during performance of the auxotonic precision grip task. D, single subject data for the average coherence in the 15.3–32.2 Hz band (yellow shading in C), using the same format at (B). There was no significant change in either tremor or coherence between the first and last half of the recorded datasets (P>0.05).

Experiments with propranolol used a double-blind placebo controlled design, as the long absorption time consequent on oral administration made it necessary to control for an effect of the time of day when recordings were made. By contrast, the rapid action of the salbutamol delivered by inhalation meant that control and drug recordings were made with only brief temporal separation. We thus chose not to make the comparison with placebo in this case. Additionally, the effects of salbutamol on heart rate and tremor were clearly apparent to the subjects, so that a double-blinded study would have been impossible. Several pieces of evidence indicated that the changes we saw following salbutamol were genuine effects of the drug, rather than reflecting progressive changes in the successive recordings. For corticomuscular coherence, Pohja et al [37] previously showed that successive measurements were highly repeatable. We also tested directly for reproducibility, by dividing each dataset recorded before salbutamol administration into two. The results from this analysis are shown in Fig. 6, for both tremor (Fig. 6AB) and corticomuscular coherence (Fig. 6CD). In neither case were there significant differences between measures made from the first versus last half of the recorded data (P>0.05). There may have been some weak trend towards reduced tremor in the later part of the recording (P = 0.14; Fig. 6B); however, even if this effect were genuine, it would be in the opposite direction to that seen following salbutamol administration (Fig. 3EF).

**Figure 7:**
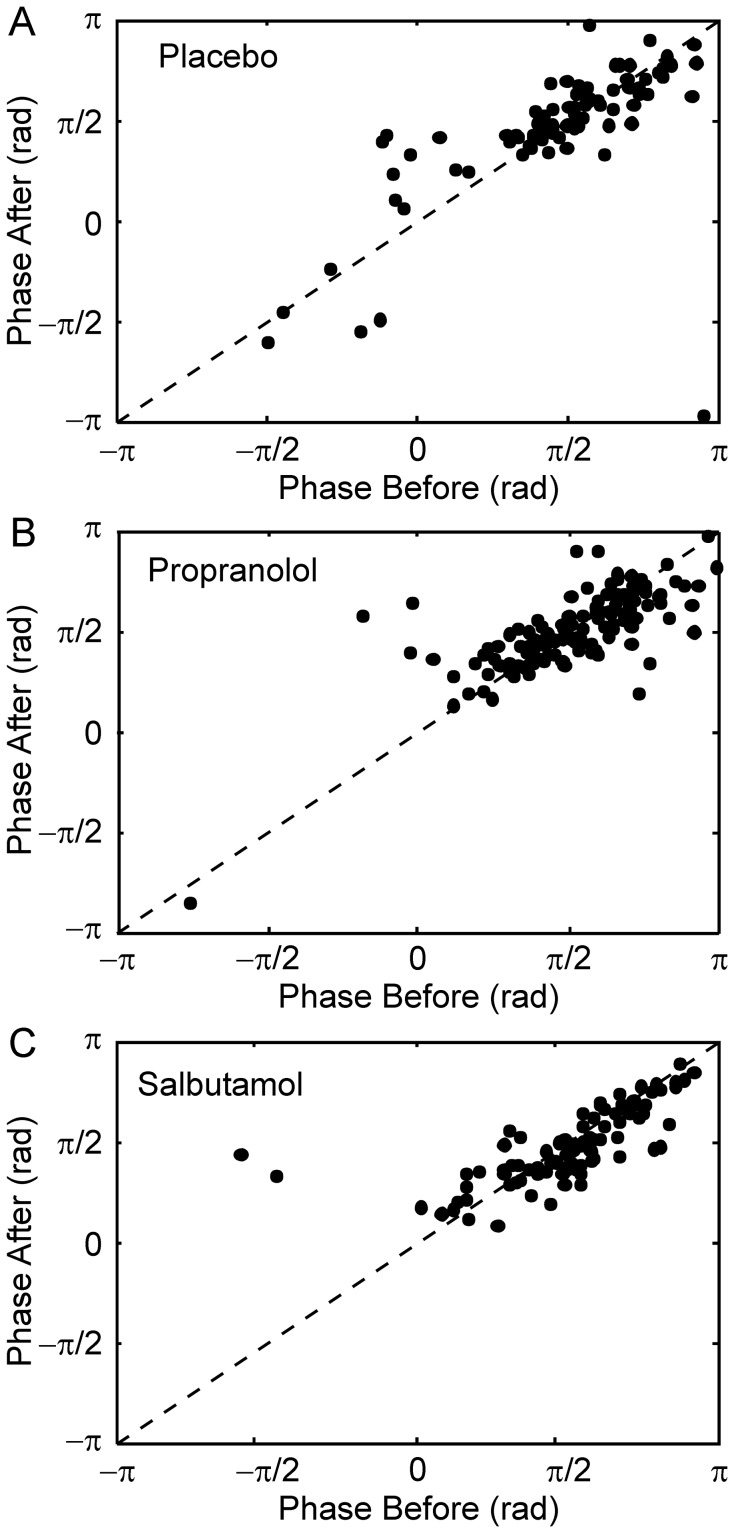
Change in Coherence Phase Following Substance Administration. Each point relates to a frequency bin in the 15.3–32.2 Hz range in individual muscles and subjects which showed significant coherence both before and after substance administration. The phase after the drug is plotted against the phase before. A, placebo, B, propranolol C, salbutamol. Dashed line indicates identity. Note that in (C) the majority of points lie below the line of identity.

Phase analysis provides important clues as to the underlying network and the likely explanation for changes in corticomuscular coherence. In order to investigate phase changes, in each EMG and each subject we found the frequency bins in the 15.3–32.2 Hz range which showed significant coherence both before and after substance administration. The phase of the coherence following administration of a substance was then plotted against the phase measured in the control experiment. Figure 7 shows the results of such an analysis, with points overlain from all EMGs and subjects. Results from placebo, propranolol, and salbutamol respectively are shown in Fig. 7A, B and C. Significant changes in phase were seen in the salbutamol dataset (circular mean phases before and after substance administration: 1.63 vs 1.51 rad, P≈0.026, Monte Carlo test). Similarly significant differences were seen when the two outlying points with negative phase before drug administration were excluded from the analysis (P≈0.017, Monte Carlo test). There were no significant changes in phase for either propranolol or placebo (both P>0.05).

## Discussion

### Differential Effects on Tremor and Corticomuscular Coherence

Surprisingly, the drugs tested in this study had different effects on ∼20 Hz corticomuscular coherence and ∼10 Hz tremor (Fig. 3 *versus* Figs. 4&5). For propranolol, the effects were reciprocal Tremor showed a weak but non-significant trend to increase at the later time of day of the second recordings with placebo (Fig. 3AC); by contrast, when propranolol was given there was a non-significant trend in the reverse direction (Fig. 3BD). Comparison of these two effects revealed a significant difference, indicating that propranolol reduced tremor compared with placebo (Fig. 3D). By contrast, propranolol elevated corticomuscular coherence (Fig. 4L). Such results are reminiscent of findings from our computational model of the effect of recurrent inhibition [29]. In this model, ∼10 Hz frequency components of the motor cortical input to motoneurones are removed by Renshaw cells, behaving as phase inverting filters, and thus ∼10 Hz tremor is reduced. By manipulating the strength of Renshaw cell recurrent inhibition, this model showed a reciprocal relationship between ∼10 Hz tremor and beta-band corticomuscular coherence (see Fig. 4 in [29]). Although the largest effect on corticomuscular coherence in the model was at 30 Hz, coherence in the entire band from 20–40 Hz was raised by increasing Renshaw cell feedback.

Whereas salbutamol clearly increased ∼10 Hz tremor (Fig. 3EF), there was no significant change in corticomuscular coherence across the subject population (Fig. 5F). It is not clear why salbutamol and propranolol failed to exert reciprocal effects on the corticomuscular coherence. This may simply be due to the low doses used, which were chosen to minimise the chances of side effects in our healthy volunteer subjects, or to β_1_-receptor or β_3_-receptor mediated effects (salbutamol is a β_2_-agonist, whereas propranolol is a non-specific β-receptor antagonist).

### Location of peripheral and central β-Adrenergic Receptors Mediating Effects

Whilst part of the action of the non-specific beta-blocker propranolol on physiological tremor could be via a peripheral site [23], [24], [28], it seems likely, in the light of our results, that there is also a central action [38]. In support of this hypothesis, propranolol, which is lipophilic, is significantly more effective at both reducing tremor [24], and penetrating the blood brain barrier [39] than the hydrophilic atenolol. Whether the central effects of propranolol are mediated via one specific class of beta-adrenergic receptor or a combination is unclear. All subtypes of beta-adrenergic receptors are widely distributed throughout the central nervous system. Immunohistochemistry has identified β_1_-adrenergic receptors in the amygdala, hippocampus, hypothalamus, cerebellum, midbrain reticular nuclei, inferior olive and striatum [40] and β_2_-adrenergic receptors in the *locus coeruleus* [42] and soma and proximal dendrites of thalamo-cortical neurones [41]. *In situ* hybridisation has further revealed β_2_-adrenergic receptors in the thalamic intralaminar nuclei, cerebellar cortex [42] and superficial dorsal horn of spinal cord [43]. β_3_-adrenergic receptor mRNA has been detected in homogenates of frontal, temporal and parietal cortex, hippocampus, striatum and midbrain by reverse transcription/polymerase chain reaction methods [44], [45].

Of the likely candidate motor areas implicated, it is unlikely that β-adrenergic effects on corticomuscular coherence are explained by actions on the cerebellum. It is known that ∼20 Hz oscillations are propagated around a loop from motor cortex to cerebellum and back [43]. However, changing the properties of this loop should change only the nature of oscillations observed in the cortex, and not alter the coupling between cortex and periphery.

Given our previous results from computational modelling, we speculate that propranolol acts centrally via Renshaw cells, producing a reciprocal modulation of ∼10 Hz tremor and 20–30 Hz corticomuscular coherence. Because Renshaw cells themselves are not known to have β-receptors, these effects would have to be mediated indirectly via inputs from neurons expressing β-adrenergic receptors, including those within the *locus coeruleus* (LC) and spinal dorsal horn. Interestingly, in the cat, Renshaw cells are in receipt of direct adrenergic reticulospinal inputs from LC, which when activated reduce recurrent inhibition [43]. Moreover, the β_3_-agonist (SR58611A) increases the firing rate of noradrenergic neurons in the *locus coeruleus* [44].

In contrast to propranolol, the striking lack of any effect of the β_2_-agoinst salbutamol upon coherence suggests that its predominant mode of action is via peripheral receptors. β_2_-adrenergic receptors are certainly found in abundance in extrafusal muscle where they mediate diverse functions including: metabolic regulation [46]; myocyte hypertrophy [47]; and repair [48]. These actions occur over a protracted time course and are therefore unlikely to be responsible for salbutamol's rapid effects on tremor. Faster processes are also mediated via β_2_-receptors. They decrease muscle excitability by closing inward rectifying potassium channels, opening calcium-activated potassium channels [49] and blocking sodium currents [50]. In addition, because sympathetic activity produces rapid changes in blood flow to skeletal muscle, it has been argued that ischaemia (or its consequences e.g. hyperkalaemia) might affect tremor by changing the biophysical properties of muscle [28].

### Network re-organization

Although the literature is conflicting [4], [51]–[54], the phase of corticomuscular coherence often does not agree with simple efferent propagation of oscillations from motor cortex to the periphery [7], [11], [19]. Rather, coherence phase probably reflects a complex interplay of feedforward and feedback pathways. In the present work, we showed that salbutamol produced a small but significant reduction in the coherence phase (Fig. 6). Although salbutamol did not change the magnitude of coherence, the reduction in phase might reflect a slight shift in the balance between feedforward and feedback pathways contributing to the coherence [11]. This could occur either by central actions of the drug, or via its previously reported peripheral actions. Beta-agonists are likely to increase the sensitivity of muscle spindles [24], whose afferents are known to encode beta-band oscillations [8].

## References

[1] Conway BA, Halliday DM, Farmer SF, Shahani U, Maas P, et al (1995) Synchronization between motor cortex and spinal motoneuronal pool during the performance of a maintained motor task in man. J Physiol 489 ((Pt 3)) 917–924. 8788955 10.1113/jphysiol.1995.sp021104 PMC1156860

[2] Murthy VN, Fetz EE (1996) Oscillatory activity in sensorimotor cortex of awake monkeys: synchronization of local field potentials and relation to behavior. J Neurophysiol 76: 3949–3967. 8985892 10.1152/jn.1996.76.6.3949

[3] Baker SN, Olivier E, Lemon RN (1997) Coherent oscillations in monkey motor cortex and hand muscle EMG show task-dependent modulation. J Physiol 501 ((Pt 1)) 225–241. 9175005 10.1111/j.1469-7793.1997.225bo.x PMC1159515

[4] Salenius S, Portin K, Kajola M, Salmelin R, Hari R (1997) Cortical control of human motoneuron firing during isometric contraction. J Neurophysiol 77: 3401–3405. 9212286 10.1152/jn.1997.77.6.3401

[5] Halliday DM, Rosenberg JR, Amjad AM, Breeze P, Conway BA, et al (1995) A framework for the analysis of mixed time series/point process data–theory and application to the study of physiological tremor, single motor unit discharges and electromyograms. Prog Biophys Mol Biol 64: 237–278. 8987386 10.1016/s0079-6107(96)00009-0

[6] Kilner JM, Baker SN, Salenius S, Hari R, Lemon RN (2000) Human cortical muscle coherence is directly related to specific motor parameters. J Neurosci 20: 8838–8845. 11102492 10.1523/JNEUROSCI.20-23-08838.2000 PMC6773054

[7] Riddle CN, Baker SN (2005) Manipulation of peripheral neural feedback loops alters human corticomuscular coherence. J Physiol 566: 625–639. 15919711 10.1113/jphysiol.2005.089607 PMC1464768

[8] Baker SN, Chiu M, Fetz EE (2006) Afferent encoding of central oscillations in the monkey arm. J Neurophysiol 95: 3904–3910. 16709725 10.1152/jn.01106.2005

[9] Baker SN (2007) Oscillatory interactions between sensorimotor cortex and the periphery. Curr Opin Neurobiol 17: 649–655. 18339546 10.1016/j.conb.2008.01.007 PMC2428102

[10] Witham CL, Wang M, Baker SN (2010) Corticomuscular coherence between motor cortex, somatosensory areas and forearm muscles in the monkey. Front Syst Neurosci 4. 10.3389/fnsys.2010.00038 PMC2927302 20740079

[11] Witham CL, Riddle CN, Baker MR, Baker SN (2011) Contributions of descending and ascending pathways to corticomuscular coherence in humans. J Physiol 589: 3789–3800. 21624970 10.1113/jphysiol.2011.211045 PMC3171886

[12] Lakie M, Walsh EG, Wright GW (1986) Passive mechanical properties of the wrist and physiological tremor. J Neurol Neurosurg Psychiatry 49: 669–676. 3734824 10.1136/jnnp.49.6.669 PMC1028850

[13] Hagbarth KE, Young RR (1979) Participation of the stretch reflex in human physiological tremor. Brain 102: 509–526. 497803 10.1093/brain/102.3.509

[14] Allum JH, Dietz V, Freund HJ (1978) Neuronal mechanisms underlying physiological tremor. J Neurophysiol 41: 557–571. 660226 10.1152/jn.1978.41.3.557

[15] Halliday DM, Conway BA, Farmer SF, Rosenberg JR (1999) Load-independent contributions from motor-unit synchronization to human physiological tremor. J Neurophysiol 82: 664–675. 10444664 10.1152/jn.1999.82.2.664

[16] Vaillancourt DE, Newell KM (2000) Amplitude changes in the 8–12, 20–25, and 40 Hz oscillations in finger tremor. Clin Neurophysiol 111: 1792–1801. 11018494 10.1016/s1388-2457(00)00378-3

[17] McAuley JH, Rothwell JC, Marsden CD (1997) Frequency peaks of tremor, muscle vibration and electromyographic activity at 10 Hz, 20 Hz and 40 Hz during human finger muscle contraction may reflect rhythmicities of central neural firing. Exp Brain Res 114: 525–541. 9187289 10.1007/pl00005662

[18] Baker SN, Pinches EM, Lemon RN (2003) Synchronization in monkey motor cortex during a precision grip task. II. effect of oscillatory activity on corticospinal output. J Neurophysiol 89: 1941–1953. 12686573 10.1152/jn.00832.2002

[19] Williams ER, Baker SN (2009a) Circuits generating corticomuscular coherence investigated using a biophysically based computational model. I. Descending systems. J Neurophysiol 101: 31–41. 19019981 10.1152/jn.90362.2008 PMC2637020

[20] Williams ER, Soteropoulos DS, Baker SN (2010) Spinal interneuron circuits reduce approximately 10-Hz movement discontinuities by phase cancellation. Proc Natl Acad Sci U S A 107: 11098–11103. 20534484 10.1073/pnas.0913373107 PMC2890710

[21] Baker MR, Baker SN (2003) The effect of diazepam on motor cortical oscillations and corticomuscular coherence studied in man. J Physiol 546: 931–942. 12563016 10.1113/jphysiol.2002.029553 PMC2342588

[22] Riddle CN, Baker MR, Baker SN (2004) The effect of carbamazepine on human corticomuscular coherence. Neuroimage 22: 333–340. 15110023 10.1016/j.neuroimage.2003.12.040

[23] Marsden CD, Foley TH, Owen DA, McAllister RG (1967) Peripheral beta-adrenergic receptors concerned with tremor. Clin Sci 33: 53–65. 6059304

[24] Abila B, Wilson JF, Marshall RW, Richens A (1985) The tremorolytic action of beta-adrenoceptor blockers in essential, physiological and isoprenaline-induced tremor is mediated by beta-adrenoceptors located in a deep peripheral compartment. Br J Clin Pharmacol 20: 369–376. 2866785 10.1111/j.1365-2125.1985.tb05079.x PMC1400891

[25] Koster B, Lauk M, Timmer J, Winter T, Guschlbauer B, et al (1998) Central mechanisms in human enhanced physiological tremor. Neurosci Lett 241: 135–138. 9507939 10.1016/s0304-3940(98)00015-9

[26] Abila B, Wilson JF, Marshall RW, Richens A (1985) Differential effects of alpha-adrenoceptor blockade on essential, physiological and isoprenaline-induced tremor: evidence for a central origin of essential tremor. J Neurol Neurosurg Psychiatry 48: 1031–1036. 2997400 10.1136/jnnp.48.10.1031 PMC1028544

[27] Raethjen J, Lemke MR, Lindemann M, Wenzelburger R, Krack P, et al (2001) Amitriptyline enhances the central component of physiological tremor. J Neurol Neurosurg Psychiatry 70: 78–82. 11118252 10.1136/jnnp.70.1.78 PMC1763454

[28] Lakie MD, Hayes NR, Combes N, Langford N (2004) Is postural tremor size controlled by interstitial potassium concentration in muscle? J Neurol Neurosurg Psychiatry 75: 1013–1018. 15201362 10.1136/jnnp.2003.022749 PMC1739132

[29] Williams ER, Baker SN (2009b) Renshaw cell recurrent inhibition improves physiological tremor by reducing corticomuscular coupling at 10 Hz. J Neurosci 29: 6616–6624. 19458232 10.1523/JNEUROSCI.0272-09.2009 PMC2690978

[30] Riddle CN, Baker SN (2006) Digit displacement, not object compliance, underlies task dependent modulations in human corticomuscular coherence. Neuroimage 33: 618–627. 16963283 10.1016/j.neuroimage.2006.07.027

[31] JointFormularyCommittee (2011) Bristish National Formulary. London: British Medical Association and Royal Pharmaceutical Society of Great Britain.

[32] Levitt DG (2002) PKQuest: a general physiologically based pharmacokinetic model. Introduction and application to propranolol. BMC Clin Pharmacol 2: 5. 12182760 10.1186/1472-6904-2-5 PMC126244

[33] Clark DJ, Tan KS, Lipworth BJ (1996) Evaluation of plasma and urinary salbutamol levels in COPD. Eur J Clin Pharmacol 51: 91–93. 8880058 10.1007/s002280050166

[34] Lipworth BJ, Clark DJ (1997) Effects of airway calibre on lung delivery of nebulised salbutamol. Thorax 52: 1036–1039. 9516895 10.1136/thx.52.12.1036 PMC1758465

[35] Evans CM, Baker SN (2003) Task-dependent intermanual coupling of 8-Hz discontinuities during slow finger movements. Eur J Neurosci 18: 453–456. 12887428 10.1046/j.1460-9568.2003.02751.x

[36] Fisher N (1993) Statistical Analysis of Circular Data. Cambridge, UK: Cambridge University Press.

[37] Pohja M, Salenius S, Hari R (2005) Reproducibility of cortex-muscle coherence. Neuroimage 26: 764–770. 15955485 10.1016/j.neuroimage.2005.02.031

[38] Zilm DH, Sellers EM (1976) The effect of propranolol on normal physiologic tremor. Electroencephalogr Clin Neurophysiol 41: 310–313. 60218 10.1016/0013-4694(76)90123-1

[39] McAinsh J, Cruickshank JM (1990) Beta-blockers and central nervous system side effects. Pharmacol Ther 46: 163–197. 1969642 10.1016/0163-7258(90)90092-g

[40] Paschalis A, Churchill L, Marina N, Kasymov V, Gourine A, et al (2009) beta1-Adrenoceptor distribution in the rat brain: an immunohistochemical study. Neurosci Lett 458: 84–88. 19442879 10.1016/j.neulet.2009.04.023

[41] Rankovic V, Landgraf P, Kanyshkova T, Ehling P, Meuth SG, et al (2011) Modulation of calcium-dependent inactivation of L-type Ca2+ channels via beta-adrenergic signaling in thalamocortical relay neurons. PLoS One 6: e27474. 22164209 10.1371/journal.pone.0027474 PMC3229489

[42] Nicholas AP, Pieribone VA, Hokfelt T (1993) Cellular localization of messenger RNA for beta-1 and beta-2 adrenergic receptors in rat brain: an in situ hybridization study. Neuroscience 56: 1023–1039. 8284033 10.1016/0306-4522(93)90148-9

[43] Nicholson R, Dixon AK, Spanswick D, Lee K (2005) Noradrenergic receptor mRNA expression in adult rat superficial dorsal horn and dorsal root ganglion neurons. Neurosci Lett 380: 316–321. 15862909 10.1016/j.neulet.2005.01.079

[44] Claustre Y, Leonetti M, Santucci V, Bougault I, Desvignes C, et al (2008) Effects of the beta3-adrenoceptor (Adrb3) agonist SR58611A (amibegron) on serotonergic and noradrenergic transmission in the rodent: relevance to its antidepressant/anxiolytic-like profile. Neuroscience 156: 353–364. 18691638 10.1016/j.neuroscience.2008.07.011

[45] Summers RJ, Papaioannou M, Harris S, Evans BA (1995) Expression of beta 3-adrenoceptor mRNA in rat brain. Br J Pharmacol 116: 2547–2548. 8590968 10.1111/j.1476-5381.1995.tb17205.x PMC1909119

[46] Duranti G, La Rosa P, Dimauro I, Wannenes F, Bonini S, et al (2011) Effects of salmeterol on skeletal muscle cells: metabolic and proapoptotic features. Med Sci Sports Exerc 43: 2259–2273. 21552152 10.1249/MSS.0b013e3182223094

[47] Hinkle RT, Hodge KM, Cody DB, Sheldon RJ, Kobilka BK, et al (2002) Skeletal muscle hypertrophy and anti-atrophy effects of clenbuterol are mediated by the beta2-adrenergic receptor. Muscle Nerve 25: 729–734. 11994968 10.1002/mus.10092

[48] Beitzel F, Gregorevic P, Ryall JG, Plant DR, Sillence MN, et al (2004) Beta2-adrenoceptor agonist fenoterol enhances functional repair of regenerating rat skeletal muscle after injury. J Appl Physiol 96: 1385–1392. 14607853 10.1152/japplphysiol.01081.2003

[49] Geukes Foppen RJ, Siegenbeek Van Heukelom J (2003) Isoprenaline-stimulated differential adrenergic response of K+ channels in skeletal muscle under hypokalaemic conditions. Pflugers Arch 446: 239–247. 12739162 10.1007/s00424-003-1042-y

[50] Desaphy JF, Pierno S, De Luca A, Didonna P, Camerino DC (2003) Different ability of clenbuterol and salbutamol to block sodium channels predicts their therapeutic use in muscle excitability disorders. Mol Pharmacol 63: 659–670. 12606775 10.1124/mol.63.3.659

[51] Brown P, Salenius S, Rothwell JC, Hari R (1998) Cortical correlate of the Piper rhythm in humans. J Neurophysiol 80: 2911–2917. 9862895 10.1152/jn.1998.80.6.2911

[52] Mima T, Steger J, Schulman AE, Gerloff C, Hallett M (2000) Electroencephalographic measurement of motor cortex control of muscle activity in humans. Clin Neurophysiol 111: 326–337. 10680569 10.1016/s1388-2457(99)00229-1

[53] Gross J, Tass PA, Salenius S, Hari R, Freund HJ, et al (2000) Cortico-muscular synchronization during isometric muscle contraction in humans as revealed by magnetoencephalography. J Physiol 527 Pt 3: 623–631. 10990546 10.1111/j.1469-7793.2000.00623.x PMC2270094

[54] Halliday DM, Conway BA, Farmer SF, Rosenberg JR (1998) Using electroencephalography to study functional coupling between cortical activity and electromyograms during voluntary contractions in humans. Neurosci Lett 241: 5–8. 9502202 10.1016/s0304-3940(97)00964-6

